# Combating Micronutrient Deficiency and Enhancing Food Functional Quality Through Selenium Fortification of Select Lettuce Genotypes Grown in a Closed Soilless System

**DOI:** 10.3389/fpls.2019.01495

**Published:** 2019-11-20

**Authors:** Antonio Pannico, Christophe El-Nakhel, Marios C. Kyriacou, Maria Giordano, Silvia Rita Stazi, Stefania De Pascale, Youssef Rouphael

**Affiliations:** ^1^Department of Agricultural Sciences, University of Naples Federico II, Portici, Italy; ^2^Department of Vegetable Crops, Agricultural Research Institute, Nicosia, Cyprus; ^3^Department of Chemical and Pharmaceutical Sciences (DSCF), University of Ferrara, Ferrara, Italy

**Keywords:** anthocyanins, carotenoids profile, hydroponics, *Lactuca* sativa L., mineral composition, nutrient solution management, phenolic acids, sodium selenate

## Abstract

Selenium (Se) is an essential trace element for human nutrition and a key component of selenoproteins having fundamental biological and nutraceutical functions. We currently examined lettuce biofortification with Se in an open-gas-exchange growth chamber using closed soilless cultivation for delivering Se-rich food. Morphometric traits, minerals, phenolic acids, and carotenoids of two differently pigmented Salanova cultivars were evaluated in response to six Se concentrations (0–40 μM) delivered as sodium selenate in the nutrient solution. All treatments reduced green lettuce fresh yield slightly (9%), while a decrease in red lettuce was observed only at 32 and 40 μM Se (11 and 21% respectively). Leaf Se content increased in both cultivars, with the red accumulating 57% more Se than the green. At 16 μM Se all detected phenolic acids increased, moreover a substantial increase in anthocyanins (184%) was recorded in red Salanova. Selenium applications slightly reduced the carotenoids content of green Salanova, whereas in red Salanova treated with 32 μM Se violaxanthin + neoxanthin, lutein and β-cryptoxanthin spiked by 38.6, 27.4, and 23.1%, respectively. Lettuce constitutes an ideal target crop for selenium biofortification and closed soilless cultivation comprises an effective tool for producing Se-enriched foods of high nutraceutical value.

## Introduction

Selenium (Se) is considered a non-essential mineral nutrient for higher plants (Sors et al., 2005; Pilon-Smits and Quinn, 2010; Malagoli et al., 2015), nevertheless several studies demonstrate the effectiveness of Se at low concentrations in improving photo-oxidative stress tolerance, delaying senescence and stimulating plant yield (Hartikainen, 2005; Lyons et al., 2009). The anti-oxidative function of Se is related to the increased activity of antioxidant enzymes including lipoxygenase, superoxide dismutase, catalase, ascorbate peroxidase, and glutathione peroxidase with the consequent decrease of lipid peroxidation, as well as to the enhanced synthesis of antioxidant molecules such as phenols, carotenoids, flavonoids, and anthocyanins in Se treated-plants (Djanaguiraman et al., 2005; Hawrylak-Nowak, 2008; Ramos et al., 2010; Ardebili et al., 2015).

While Se is considered merely beneficial to plants (Pilon-Smits et al., 2009; Vatansever et al., 2017; Chauhan et al., 2019), it is deemed essential for animal and human nutrition as it constitutes the key component of selenoenzymes and selenoproteins with fundamental biological functions (Rayman, 2002). Low dietary intake of Se has been associated with serious human illnesses, such as cardiovascular diseases, viral infections and certain types of cancer (Rayman, 2000; Combs, 2001; Finley, 2005). Selenium deficiency has been estimated to affect up to one billion people worldwide (Jones et al., 2017). Most serious consequences have been reported in China, the UK, Eastern Europe, Africa, and Australia (Chen et al., 2002; Lyons et al., 2004), in areas with arable soils of low Se bioavailability that inevitably limits Se entry into the food supply chain.

The Recommended Dietary Allowance (RDA) of Se for adult men and women is 55 µg day^−1^ (Johnson et al., 2003), however, Burk et al. (2006) have found that Se supplementation of 200 µg day^−1^, reduces the risk of prostate, lung and colon cancer. Plants constitute a potentially significant source of this element for human diet through biofortification. Biofortification is the process that increases the bioavailable content of targeted elements in edible plant parts through agricultural intervention or genetic selection (White and Broadley, 2005). In this perspective, recent works have demonstrated that Se fertilization increases the content of this element in a wide range of crops including rice (Chen et al., 2002), wheat (Lyons et al., 2004), radish (Pedrero et al., 2006; Schiavon et al., 2016), spinach (Ferrarese et al., 2012), potato (Turakainen et al., 2004), bean (Hermosillo-Cereceres et al., 2011), soybean (Yang et al., 2003), pea (Jerše et al., 2018), tomato (Schiavon et al., 2013), rocket (Dall’Acqua et al., 2019), lamb’s lettuce (Hawrylak-Nowak et al., 2018), and lettuce (Businelli et al., 2015; Esringu et al., 2015; Smolen et al., 2016a; Silva et al., 2017; Silva et al., 2018a). Se fertilization is a relatively low-cost approach to the prophylaxis of consumers against nutrient deficiency. Several countries, such as Finland, Malawi, Australia, and New Zealand, have supported this strategy through biofortification programs, demonstrated to boost Se content in human tissue and body fluids of the population (Arthur, 2003; Eurola et al., 2004; Chilimba et al., 2012), as well as Brazil, where studies were performed on upland rice (Reis et al., 2018), rice (Andrade et al., 2018) and cowpea (Silva et al., 2018b; Silva et al., 2019).

Higher plant roots uptake Se mainly as selenate and selenite. Selenate is transported across the plasma membrane of root cells, using the assimilation pathways of sulfate *via* the enzyme sulfate permease (Terry et al., 2000; Hawkesford and Zhao, 2007), while selenite is transported *via* phosphate transporters (Li et al., 2008). The selectivity of these transporters is species-dependent and affected by soil sulfate concentration, salinity, pH and redox potential (Combs, 2001; White et al., 2004); moreover, the different types of sulphate transporters (SULTR1;1, SULTR1;2, SULTR2;1) may have different selectivity for selenium and sulfur (Dall’Acqua et al., 2019). Nevertheless, selenate is more soluble, less phytotoxic and easily transported and accumulated in crops compared to selenite (Lyons et al., 2005; Smrkolj et al., 2005; Hawrylak-Nowak, 2013).

Regarding the bioactive value of Se, several studies have demonstrated its role in plant secondary metabolism by increasing tocopherol, flavonoids, phenolic compounds, ascorbic acid and vitamin A (Hartikainen et al., 2000; Xu et al., 2003; Ríos et al., 2008; Businelli et al., 2015), noting that plant secondary metabolites are health promoting phytochemicals that prevent a range of human diseases and are used as well as medicinal active ingredients (El-Nakhel et al., 2019). However, at high concentrations Se is phytotoxic, inhibiting growth and modifying the nutritional characteristics of plants (Hartikainen et al., 2000). Selenium phytotoxicity is attributable to non-specific incorporation of selenocysteine (SeCys) and selenomethionine (SeMet) which replace their sulphur analogues compounds in plant proteins (Ellis and Salt, 2003).

Vegetables are widely used in biofortification studies, including lettuce (*Lactuca sativa* L.), which is the most produced and consumed leafy vegetable in the world (Baslam et al., 2013; Hawrylak-Nowak, 2013). It has attained a central role in human nutrition as it combines palatable organoleptic properties with a rich content of nutraceutical compounds (phenolic acids, carotenoids, flavonoids, and vitamins B9, C, and E) and a low content of dietary fats, which makes lettuce an attractive low-calorie food (Kim et al., 2016). Moreover, since lettuce is generally eaten raw, more nutrients are retained compared to cooked foods, including Se that has been has been shown to diminish in concentration after food processing, such as boiling, baking or grilling (Dumont et al., 2006; Sager, 2006). Being also one of the most easily cultivated vegetables both in soil and in hydroponic systems, lettuce can be considered therefore a promising candidate for Se biofortification.

Several biofortification techniques have been proposed, such as soil/substrate dosing with Se, foliar spray with Se solution and hydroponic cultivation with Se enriched nutrient solution (Smrkolj et al., 2007; Puccinelli et al., 2017; Wiesner-Reinhold et al., 2017). The technique choice should consider, among other aspects, the possible run-off of Se fertilizers resulting in Se accumulation in groundwater. In this respect, hydroponic cultivation, especially in closed-loop systems, has several advantages: (i) environmental spread of Se is minimized, (ii) Se uptake is higher than other methods, as the constant exposure of the roots with the fortified nutrient solution and the absence of micronutrient-soil interactions maximize uptake efficiency and accumulation in edible plant parts, (iii) product quality is standardized through precise management of the concentration and composition of nutrient solution, (iv) very small amounts of selenium are needed, and no modification of conventional closed soilless cultivation technique is required thus ensuring no additional cost (Puccinelli et al., 2017; Wiesner-Reinhold et al., 2017; Rouphael and Kyriacou, 2018).

Taking into account these considerations, the effects of sodium selenate application were evaluated in this present work at six different doses on two lettuce cultivars of different pigmentation (green and red) cultivated in a closed soilless system. The aim of this study was to identify the appropriate Se concentration in the nutrient solution in order to maximize the accumulation of selenium and enhance the nutraceutical characteristics (lipophilic and hydrophilic antioxidant molecules), by creating a dual enrichment of lettuce, without causing important loss of yield in lettuce.

## Materials and Methods

### Growth Chamber Conditions, Plant Material and Experimental Design

Two butterhead lettuce (*L. sativa* L. var. capitata) cultivars with different leaf pigmentation, green Salanova^®^ “Descartes” and red Salanova^®^ “Klee” (Rijk Zwaan, Der Lier, The Netherlands), were cultivated in a 28 m^2^ open-gas-exchange growth chamber (7.0 m × 2.1 m × 4.0 m, width × height × depth) situated at the experimental station of the University of Naples Federico II, Italy.

The lighting of the growth chamber was provided by High Pressure Sodium lamps (Master SON-T PIA Plus 400W, Philips, Eindhoven, The Netherlands) with a photosynthetic photon flux density (PPFD) of 420 ± 10 µmol m^−2^ s^−1^, measured at leaf height using a spectral radiometer (MSC15, Gigahertz-Optik, Turkenfeld, Germany). Day/night temperatures of 24/18°C were established with a 12 h photoperiod and a relative air humidity of 60−80% respectively. The experiment was carried out at ambient CO_2_ concentration (390 ± 20 ppm), while air exchange and dehumidification were guaranteed by two HVAC systems. Plants were grown in nutrient film technique (NFT) established on rigid polyvinyl chloride (PVC) gullies (14.5 cm wide, 8 cm deep and 200 cm long), with a 1% slope. The gullies were 60 cm above ground level and each of them was fed by a separate 25 L plastic reservoir tank containing the nutrient solution (NS). Continuous recirculation (1.5 L min^−1^) of the NS was provided by a submerged pump (NJ3000, Newa, Loreggia, PD, Italy) in each reservoir tank. Twenty-day-old lettuce seedlings were transplanted in rockwool cubes (7 × 7 × 7cm, Delta, Grodan, Roermond, The Netherlands) and transferred into the gullies with an intra-row and inter-row spacing of 15 and 43 cm respectively, corresponding to a density of 15.5 plants m^−2^. Each gully was covered with PVC lid in order to avoid NS evaporation. The NS was a modified Hoagland formulation prepared with osmotic water containing: 8.0 mM N–NO_3_^−^, 1.5 mM S, 1.0 mM P, 3.0 mM K, 3.0 mM Ca, 1.0 mM Mg, 1.0 mM NH_4_^+^, 15 µM Fe, 9 µM Mn, 0.3 µM Cu, 1.6 µM Zn, 20 µM B, and 0.3 µM Mo, with electrical conductivity (EC) 1.4 dS m^−1^ and pH 6.0 ± 0.1.

The experimental design was a randomized complete-block factorial design (6 × 2) with six selenium concentrations in the nutrient solution (0, 8, 16, 24, 32, or 40 µM as sodium selenate, from Sigma-Aldrich, St. Louis, MO, USA) and two lettuce cultivars (green or red butterhead Salanova), with three replicates. Each experimental plot consisted of six plants.

### Growth Analysis and Biomass Determination

Plants were harvested at nineteen days after transplant (DAT). Number of leaves and fresh weight of the aerial plant parts were determined, then leaf area was measured by an area meter (LI-COR 3100C, Biosciences, Lincoln, Nebraska, USA).

Leaf dry weight was determined on an analytical balance (Denver Instruments, Denver, Colorado, USA) after sample desiccation in a forced-air oven at 70°C to constant weight (around 72 h). Leaf dry matter was determined according to the official method 934.01 of the Association of Official Analytical Chemists.

### Collection of Samples for Mineral and Nutritional Quality Analyses

Part of the dried leaf tissue of green and red Salanova plants was used for macro-mineral and selenium analyses. For the identification and quantification of phenolic acids and carotenoid compounds by HPLC-DAD, fresh samples of three plants per experimental unit were instantly frozen in liquid nitrogen and stored at −80°C before lyophilizing them in a Christ, Alpha 1–4 (Osterode, Germany) freeze drier.

### Mineral Analysis by Ion Chromatography and ICP-OES and Consumer Safety of Se-Enriched Butterhead Lettuce

Leaf soluble cations and anions were determined by liquid ion exchange chromatography (ICS 3000 Dionex Sunnyvale, CA, USA) with conductimetric detection, as described previously by Rouphael et al. (2017b). Briefly, 250 mg of dried sample ground at 0.5 mm in a Wiley Mill (IKA, MF 10.1, Staufen, Germany) were suspended in 50 ml of ultrapure water (Milli-Q, Merck Millipore, Darmstadt, Germany) and stirred in shaking water bath (ShakeTemp SW22, Julabo, Seelbach, Germany) at 80° C for 10 min. The mixture was centrifuged at 6,000 rpm for 10 min (R-10M, Remi Elektrotechnik Limited, India), then filtered through a 0.45 µm syringe filter (Phenomenex, Torrance, CA, USA). Chromatographic separation of Na, K, Mg, and Ca was achieved in isocratic mode (20 mM methanesulphonic acid) on an IonPac CS12A analytical column (4 × 250 mm, Dionex Sunnyvale, CA, USA) equipped with an IonPac CG12A precolumn (4 × 250 mm, Dionex Sunnyvale, CA, USA) and a self-regenerating suppressor CERS500 (4 mm, Dionex Sunnyvale, CA, USA). Nitrate, phosphate, and sulphate were detected in gradient mode (1mM-50mM KOH) on an IonPac ATC-HC anion trap (9×75 mm, Dionex Sunnyvale, CA, USA), and an AS11-HC analytical column (4 × 250 mm, Dionex Sunnyvale, CA, USA) equipped with an AG11-HC precolumn (4 × 50 mm, Dionex Sunnyvale, CA, USA) and a self-regenerating suppressor AERS500 (4 mm, Dionex Sunnyvale, CA, USA). Ions were expressed as g kg^−1^ dry weight (dw) and nitrate was expressed as mg kg^−1^ fresh weight (fw) on the basis of each sample’s original dw.

In addition to macro-minerals analysis, Se content was also measured in green and red Salanova leaf tissue. Each sample was subjected to a first phase of acid digestion performed using a commercial high-pressure laboratory microwave oven (Mars plus CEM, Italy) operating at an energy output of 1,800 W. Approximately 300 mg of each dry sample was inserted directly into a microwave-closed vessel. Two milliliters of 30% (m/m) H_2_O_2_, 0.5 ml of 37% HCl and 7.5 ml of HNO_3_ 69% solution were added to each vessel. The heating program was performed in one step: temperature was ramped linearly from 25 to 180°C in 37 min, then held at 180°C for 15 min. After the digestion procedure and subsequent cooling, samples were transferred into a Teflon beaker and total volume was made up to 25 ml with Milli-Q water. The digest solution was then filtered through DISMIC 25HP PTFE syringe filter of pore size 0.45 mm (Toyo Roshi Kaisha, Ltd., Japan) and stored in a screw cap plastic tube (Nalgene, New York). Blanks were prepared in each lot of samples. All experiments were performed in triplicate. The reagents of super pure grade, used for the microwave-assisted digestions, were: hydrochloric acid (36% HCl), nitric acid (69% HNO_3_), and hydrogen peroxide (30% H_2_O_2_) (Merck, Darmstadt, Germany). High-purity water (18 MΩcm^−1^) from a Milli-Q water purification system (Millipore, Bedford, USA) was used for the dilution of the standards, for preparing samples throughout the chemical process, and for final rinsing of the acid-cleaned vessels, glasses, and plastic utensils. For this work, tomato leaves (SRM 1573a) were used as external certified reference material. Selenium quantification was performed using an Inductively Coupled Plasma Optical Emission Spectrometer (ICP-OES) with an axially viewed configuration (8,000 DV, PerkinElmer, Shelton, CT, USA) equipped with an Hydride Generation system for Se quantification at 196.06 nm. Twenty-five ml of digested material was pre-reduced by concentrated HCl (5 ml, superpure grade) followed by heating at 90°C for 20 min. After pre-reduction, the solution was diluted to 50 ml in polypropylene vial with deionized water (18 MΩ cm^−1^). In order to determine the Se concentration calibration standards were prepared, treated in same way before dilution. Selenium content in lettuce leaves was expressed as mg kg-1 dw.

The green vegetables hazard quotient (HQ_gv_) was calculated according to the United States Environmental Protection Agency (USEPA) Protocol using the following formula:

$$\text{H}{\text{Q}}_{\text{gv}}=(\text{ADD}/\text{RfD})$$

where ADD is the average daily dose of selenium (µg Se day^−1^) and RfD represents the recommended dietary tolerable upper intake level of selenium (µg Se day^−1^) assessed equal to 400 µg day^−1^ (Johnson et al., 2003), referring to the risk to human health of a 70-kg adult resulting from Se intake through the consumption of a 50-g portion of fresh lettuce.

### Phenolic Acids and Anthocyanins Identification and Quantification

Four hundred mg of lyophilized samples were solubilized in a solution of methanol/water/formic acid (50/45/5, v/v/v, 12 ml) as described by Llorach et al. (2008) to determine phenolic acids as hydroxycinnamic derivatives. The suspensions were sonicated for 30 min and then subjected to centrifugation (2,500 *g* for 30 min at 4°C). After a second centrifugation of supernatants at 21,100 *g* for 15 min at 4°C, samples were filtered through 0.22 µm cellulose filters (Phenomenex). A reversed phase C18 column (Prodigy, 250 × 4.6 mm, 5 µm, Phenomenex, Torrance, CA) equipped with a C18 security guard (4.0 × 3.0 mm, Phenomenex) was utilized for the separation of hydroxycinnamic derivatives and anthocyanins. Twenty µL of each extract were injected and the following elution gradient was built based on solvent (A) water formic acid (95:5, v/v) and (B) methanol: (0/5), (25/40), (32/40) in min/%B. The flow rate was 1 ml min^−1^. The LC column was installed onto a binary system (LC-10AD, Shimadzu, Kyoto, Japan), equipped with a DAD (SPD-M10A, Shimadzu, Kyoto, Japan) and a Series 200 autosampler (Perkin Elmer, Waltham, MA). Chlorogenic and chicoric acids at 330 nm were used for the calibration curves of hydroxycinnamic derivatives. Identification of caffeoyl-meso-tartaric acid and caffeoyl-tartaric acid was performed by LC-MS/MS experiments.

The chromatographic profiles of reference curves and samples were recorded in multiple reaction monitoring mode (MRM) by using an API 3000 triple quadrupole (ABSciex, Carlsbad, CA). Negative electrospray ionization was used for detection and source parameters were selected as follows: spray voltage −4.2 kV; capillary temperature: 400°C, dwell time 100 ms, nebulizer gas and cad gas were set to 10 and 12 respectively (arbitrary units). Target compounds [M–H]− were analyzed using mass transitions given in parentheses: chicoric acid (m/z 473 311, 293), chlorogenic acid (m/z 353 191), caffeoyl tartaric acid (m/z 311 179, 149, retention time 15.8 min), caffeoyl-meso-tartaric acid (m/z 311 179, 149, retention time 17.8 min). The concentration of phenolic acids was reported as mg 100 g^−1^ of dw.

Anthocyanins were also measured within the same LC-DAD chromatographic runs, at 520 nm and the concentration calculated by using cyanidin as reference standard to calculate the concentration. The results were reported as µg of cyanidin equivalent per g of dw.

### Carotenoids Identification and Quantification

One gram of lyophilized samples was used to determine carotenoids content following the method of Vallverdú-Queralt et al. (2013) with slight modifications. Samples were solubilized in ethanol/hexane (4:3, v/v, 2.5 ml) with 1% BHT, vortexed at 22°C for 30 s and sonicated for 5 min in the dark. Then, the solution was centrifuged (2500 g, 4°C, 10 min) and filtered through 0.45 µm nylon syringe filters (Phenomenex, Torrance, CA, USA). The extracts were dried in N and the dried extracts were dissolved in 1% BHT in chloroform. Twenty µl of each sample was injected onto a C18 column (Prodigy, 250 × 4.6 mm, 5 µm, Phenomenex, Torrance, C A, USA) with a C18 security guard (4.0 × 3.0 mm, Phenomenex). Two mobile phases were used: (A) acetonitrile, hexane, methanol, and dichloromethane (4:2:2:2, v/v/v/v) and (B) acetonitrile. Carotenoids were eluted at 0.8 ml min^−1^ through the following gradient of solvent B (t in [min]/[%B]): (0/70), (20/60), (30/30), (40/2). Carotenoids were quantified by a binary LC-10AD system connected to a DAD (SPD-M10A, Shimadzu, Kyoto, Japan) equipped with a Series 200 auto-sampler (Perkin Elmer, Waltham, MA, USA). Violaxanthin, neoxanthin, β-cryptoxanthin, lutein and β-carotene were used as reference standards. Identification of the peaks was achieved by comparison of UV-vis spectra and retention times of eluted compounds with pure standards at 450 nm. Three separate sets of calibration curves were built; each set was injected three times in the same day (intraday assay) and three times in three different days (interday assay). The accuracy was reported as the discrepancies between the calibration curves performed intraday and interday and the results were expressed as relative standard deviation RSD (%). A recovery test was performed spiking two samples with two known amounts of carotenoids (50 and 100 µg ml^−1^ final concentration) and taking into account the overestimation due to the target analytes already present in the samples. Except for violaxanthin + neoxanthin which was expressed as µg violaxanthin equivalent per g dw, the concentration of the target carotenoids was expressed as µg g−1 of dw.

### Statistics

All morphometric, nutritional and functional quality data were subjected to analysis of variance (two-way ANOVA) using IBM SPSS 20 software package (www.ibm.com/software/analytics/spss). Cultivar means were compared by t-Test. Duncan’s multiple range test was performed for comparisons of the selenium treatment means. In order to determine the interrelationship among the morphometric, nutritional and functional quality traits in respect to the experimental treatments, a Principal Component Analysis (PCA) was performed using the appropriate function PCA from the SPSS 20 software package.

## Results and Discussion

### Advanced Integrative Simultaneous Analysis of Morpho-Physiological Traits

Genetic material is the main pre-harvest factor that strongly affects the biometric characteristics as well as the biosynthesis, the composition and accumulation of bioactive compounds (Kim et al., 2016). For most of the measured agronomic parameters no significant interaction between the two tested factors, lettuce cultivar (C) and Se concentration in the nutrient solution (Se), was recorded, except for leaf area and fresh yield (**Table 1**). In particular, green Salanova had higher leaf number, shoot dry biomass and leaf dry matter content (%). Regarding the effect of Se concentration in the nutrient solution, increasing Se concentration to 24 µM resulted in non-significant differences in shoot dry biomass with the control (0 µM) and 16 µM treatments; whereas increasing Se concentration from 0 to 40 µM yielded a significant increase in leaf dry matter content, with the highest values observed at 40 µM (5.7%) (**Table 1**). Leaf number was not affected by the addition of Se to the nutrient solution.

**Table 1:** Growth parameters, fresh biomass, dry biomass and leaf dry matter content of green and red Salanova lettuce grown hydroponically in a Fitotron open-gas-exchange growth chamber under six Se concentrations applied in the nutrient solution.

Source of variance	Leaf area (cm^2^ plant^−1^)	Leaf number (no. plant^−1^)	Fresh biomass (g plant^−1^)	Dry biomass (g plant^−1^)	Dry matter (%)
Cultivar (C)					
Green Salanova	1,193 ± 16.5	59 ± 0.79 a	78.55 ± 1.13	4.32 ± 0.05 a	5.48 ± 0.06 a
Red Salanova	1,147 ± 21.8	55 ± 0.69 b	76.95 ± 1.65	3.96 ± 0.06 b	5.19 ± 0.06 b
t-test	ns	***	ns	***	***
Selenium (µM Se) (S)					
0	1,253 ± 27.8	57 ± 1.26	84.33 ± 1.71	4.26 ± 0.15 ab	5.06 ± 0.07 d
8	1,141 ± 18.0	56 ± 1.37	76.69 ± 1.47	4.04 ± 0.06 b	5.28 ± 0.06 bc
16	1,192 ± 25.6	57 ± 1.46	80.04 ± 0.95	4.15 ± 0.08 ab	5.18 ± 0.10 cd
24	1,186 ± 8.3	57 ± 1.02	80.46 ± 1.84	4.37 ± 0.06 a	5.33 ± 0.08 bc
32	1,121 ± 37.7	56 ± 2.15	74.87 ± 1.46	4.03 ± 0.13 b	5.44 ± 0.06 b
40	1,127 ± 49.8	60 ± 2.23	70.09 ± 2.35	4.01 ± 0.19 b	5.71 ± 0.09 a
	**	ns	***	*	***
C x S					
Green Salanova × 0 µM Se	1,207 ± 29.6 ab	59 ± 1.02	86.29 ± 1.47 a	4.48 ± 0.12	5.19 ± 0.07
Green Salanova × 8 µM Se	1,126 ± 21.2 bcd	58 ± 0.85	75.72 ± 2.88 cd	4.07 ± 0.10	5.38 ± 0.07
Green Salanova × 16 µM Se	1,236 ± 22.6 ab	59 ± 1.76	79.30 ± 1.85 bcd	4.26 ± 0.10	5.38 ± 0.09
Green Salanova × 24 µM Se	1,201 ± 6.2 ab	57 ± 1.02	78.08 ± 1.71 bcd	4.48 ± 0.02	5.50 ± 0.02
Green Salanova × 32 µM Se	1,169 ± 66.9 bc	58 ± 3.00	76.90 ± 2.42 bcd	4.23 ± 0.20	5.53 ± 0.10
Green Salanova × 40 µM Se	1,219 ± 59.8 ab	64 ± 0.93	74.99 ± 0.97 cd	4.41 ± 0.09	5.88 ± 0.12
Red Salanova × 0 µM Se	1,299 ± 29.5 a	55 ± 1.19	82.37 ± 2.93 ab	4.05 ± 0.23	4.94 ± 0.08
Red Salanova × 8 µM Se	1,157 ± 30.4 bc	53 ± 1.47	77.67 ± 1.26 bcd	4.01 ± 0.08	5.17 ± 0.06
Red Salanova × 16 µM Se	1,147 ± 27.5 bc	55 ± 1.35	80.78 ± 0.76 abc	4.03 ± 0.11	4.99 ± 0.09
Red Salanova × 24 µM Se	1,172 ± 9.3 bc	57 ± 2.04	82.84 ± 2.87 ab	4.27 ± 0.08	5.17 ± 0.09
Red Salanova × 32 µM Se	1,074 ± 18.1 cd	53 ± 2.92	72.84 ± 0.86 d	3.82 ± 0.05	5.35 ± 0.05
Red Salanova × 40 µM Se	1,036 ± 21.8 d	55 ± 1.11	65.20 ± 1.67 e	3.61 ± 0.07	5.54 ± 0.03
	**	ns	*	ns	ns

ns,*,**, *** Non-significant or significant at P ≤ 0.05, 0.01, and 0.001, respectively. In the absence of interaction, cultivar means were compared by t-Test and Se application means by Duncan’s multiple-range test (P = 0.05). Different letters within each column indicate significantly different means. All data are expressed as mean ± SE, n = 3.

Leaf area and fresh biomass incurred significant interaction of the tested factors (**Table 1**), as the dose effect of Se on these two morphometric traits was cultivar-dependent. In the red cultivar, a reduction of the leaf area was observed with increasing Se dose, amounting to about 11% reduction in the range of 8–32 µM Se and up to 19% at the higher Se dose (40 µM) compared to the control treatment; whereas no significant differences were recorded in the green cultivar. Cultivars/genotypes may develop different Se-tolerance and response mechanisms depending on the concentration and time of exposure. This was the case in the current experiment, since fresh yield decreased in both cultivars with increasing Se concentration in the nutrient solution although the red-pigmented butterhead lettuce was less affected than the green-pigmented cultivar especially at mild and moderate Se concentrations (i.e. 8 to 24 µM) (**Table 1**). In red Salanova, fresh yield was not affected by the addition of Se up to a concentration of 24 µM, whereas the addition of 32 µM and especially 40 µM induced a reduction in the fresh biomass of 11 and 21%, respectively, compared to the 0, 8, 16, and 24 µM treatments. Finally, a significant decrease in green Salanova fresh biomass (about 10%) was observed in response to Se application without significant differences between the five Se treatments (**Table 1**).

Several studies demonstrate the beneficial or toxic effects on morphometric traits of lettuce depending on the interaction of cultivar and application level (Ríos et al., 2008; Rios et al., 2010a; Ramos et al., 2011; Hawrylak-Nowak, 2013). Ramos et al. (2011) studied the influence of 15 µM of selenate and 15 µM of selenite concentrations in the nutrient solution on the yield of 30 lettuce accessions grown hydroponically. The authors reported that just 5 of 30 accessions treated with 15 µM of selenate showed an increase in fresh biomass compared to the control. Contrarily, Hawrylak-Nowak (2013) confirmed a decrease in both leaf area and fresh biomass of green lettuce cv. Justyna grown hydroponically and supplied with 10 µM of selenate, while in another similar work on green lettuce cv. Vera, a reduction of dry biomass was observed only at 8 µM selenate dose (Ramos et al., 2010), both of which findings are in line with our current ones on green Salanova. Additional studies conducted by Ríos et al. (2008; 2010a) also reported a decrease of dry biomass in hydroponically grown green lettuce (cv. Philipus) treated continuously with nutrient solution containing 80 µM Se compared to the control treatment.

The cultivar-dependent response to supplemental Se observed in our experiment, where the red-pigmented Salanova showed better tolerance to selenate compared to the green one, was in agreement with the study on red lettuce cv. Veneza Roxa by Silva et al. (2018a), where no signiﬁcant reduction in shoot fresh weight was observed with selenate concentrations ranging from 10 to 40 µM. Considering the above, it appears that the beneficial or toxic effect of Se on plant growth and crop productivity may vary in relation to different interacting variables, including the Se concentration, time of exposure and cultivation system (Pedrero and Madrid, 2009). In the light of this finding, additional studies should focus on elucidating the cultivar × application dose × cultivation system (soilless versus soil) interaction in order to select optimal combinations to ensure balance between yield and biofortification.

### Nitrate Content, Mineral Composition, Selenium Biofortification, and Consumer Safety

Nitrate content in plants grown for human consumption is extremely important, since a high intake of this nutrient may harm human health due to its potential transformation to nitrite and nitrogenous compounds that can cause serious pathological disorders, such as methaemoglobinaemia and blue baby syndrome (Colla et al., 2018). In addition, it should be taken into account that lettuce is considered a nitrate hyper-accumulator; hence the European Commission (Commission Regulation no. 1258/2011) has set as maximum limit for nitrate concentration in lettuce at 4,000 and 5,000 mg kg^−1^ fw for harvest occurring from April 1 to September 30 and from October 1 to March 31, respectively. In respect to the effect of Se concentration in the nutrient solution, the green cultivar had a higher nitrate content (1,810 mg kg^−1^ fw) than the red one (1,272 mg kg^−1^ fw), however both values were by far below EU regulation limits (**Table 2**). In fact, it is well established that nitrate accumulation in lettuce, aside from the cultivation management, depends mainly on genotypic factors (Burns et al., 2010; Burns et al., 2011; López et al., 2014). In the current study, nitrate content was influenced by both tested factors and the cultivar × Se interaction (**Table 2**). In green Salanova a significant reduction of nitrate content was observed at 8 µM (15%), 32 µM (16%), and 40 µM Se (32%) compared to the control, while no significant Se effect was found regarding this parameter in red Salanova (**Table 2**). The reduction of nitrate content prompted by selenate could be associated to the antagonistic relation of these two anions (Rios et al., 2010a). Moreover, Nowak et al. (2004) have demonstrated that Se affects the nitrate reductase enzyme, increasing its activity in plants. In addition, the reduction in foliar nitrate could be related to a greater assimilation rate of this anion due to a higher amino acid synthesis driven by enhanced nitrate reductase activity. In fact, Se toxicity in plants may be due to the formation of non-specific selenoproteins; in particular, the replacement of cysteine (Cys) with SeCys in non-specific selenoproteins would invoke a higher demand of amino acids for the synthesis of functional proteins, which would elicit the removal of these malformed selenoproteins (Van Hoewyk, 2013). Our data reflect a nitrate reduction observed in previous works, where selenate has been applied on green-pigmented lettuce at different concentrations (Lee et al., 2008; Rios et al., 2010a; Ríos et al., 2010b).

**Table 2:** Nitrate, phosphate, sulphate, potassium (K), calcium (Ca), magnesium (Mg) and sodium (Na) concentrations of green and red Salanova lettuce grown hydroponically in a Fitotron open-gas-exchange growth chamber under six Se concentrations applied in the nutrient solution.

Source of variance	Nitrate (mg kg^−1^ fw)	Phosphate (g kg^−1^ dw)	Sulphate (g kg^−1^ dw)	K (g kg^−1^ dw)	Ca (g kg^−1^ dw)	Mg (g kg−1dw)	Na (g kg−1 dw)
Cultivar (C)							
Green Salanova	1,810 ± 69	14.9 ± 0.37	5.7 ± 0.93	59.50 ± 1.19	6.13 ± 0.09 a	2.25 ± 0.03	0.36 ± 0.012
Red Salanova	1,272 ± 25	14.3 ± 0.37	14.8 ± 2.31	54.81 ± 0.67	5.21 ± 0.11 b	2.62 ± 0.04	0.39 ± 0.029
t-test	***	ns	***	**	***	***	ns
Selenium (µM Se) (S)							
0	1,660 ± 175	16.3 ± 0.55 a	2.9 ± 0.36	58.57 ± 3.00	5.73 ± 0.35 a	2.41 ± 0.06	0.37 ± 0.039
8	1,480 ± 112	15.5 ± 0.21 ab	3.9 ± 0.63	54.75 ± 1.12	5.62 ± 0.14 ab	2.31 ± 0.06	0.32 ± 0.010
16	1,680 ± 149	15.5 ± 0.06 ab	6.5 ± 1.38	58.71 ± 1.72	6.00 ± 0.29 a	2.52 ± 0.10	0.36 ± 0.013
24	1,704 ± 168	14.7 ± 0.15 b	10.5 ± 2.67	60.04 ± 1.56	5.68 ± 0.18 a	2.47 ± 0.11	0.35 ± 0.011
32	1,487 ± 111	13.1 ± 0.43 c	17.7 ± 4.14	58.18 ± 1.19	5.80 ± 0.23 a	2.51 ± 0.13	0.44 ± 0.076
40	1,234 ± 64	12.5 ± 0.28 c	20.0 ± 3.12	52.69 ± 0.84	5.21 ± 0.28 b	2.39 ± 0.13	0.42 ± 0.033
	***	***	***	*	*	*	ns
C x S							
Green Salanova × 0 µM Se	2,011 ± 168 a	16.9 ± 1.01	2.1 ± 0.24 f	63.49 ± 4.54 a	6.34 ± 0.37	2.40 ± 0.11 bc	0.44 ± 0.043 bc
Green Salanova × 8 µM Se	1,718 ± 68 b	15.3 ± 0.37	2.5 ± 0.06 f	56.89 ± 0.69 cd	5.86 ± 0.14	2.17 ± 0.03 d	0.34 ± 0.005 cd
Green Salanova × 16 µM Se	2,011 ± 30 a	15.5 ± 0.06	3.4 ± 0.18 ef	62.52 ± 0.36 ab	6.49 ± 0.23	2.31 ± 0.06 bcd	0.37 ± 0.023 bcd
Green Salanova × 24 µM Se	2,074 ± 46 a	14.9 ± 0.31	4.5 ± 0.09 e	63.38 ± 0.94 a	6.04 ± 0.10	2.22 ± 0.04 cd	0.35 ± 0.017 cd
Green Salanova × 32 µM Se	1,681 ± 148 b	13.7 ± 0.74	9.4 ± 0.45 d	57.83 ± 1.56 bcd	6.29 ± 0.08	2.22 ± 0.02 cd	0.31 ± 0.011 d
Green Salanova × 40 µM Se	1,366 ± 36 c	12.9 ± 0.21	12.3 ± 1.05 c	52.91 ± 1.15 d	5.74 ± 0.07	2.15 ± 0.03 d	0.36 ± 0.016 bcd
Red Salanova × 0 µM Se	1,309 ± 36 cd	15.7 ± 0.34	3.6 ± 0.18 ef	53.66 ± 0.39 cd	5.11 ± 0.30	2.42 ± 0.07 bc	0.29 ± 0.010 d
Red Salanova × 8 µM Se	1,242 ± 41 cd	15.6 ± 0.34	5.3 ± 0.15 e	52.62 ± 1.10 d	5.37 ± 0.12	2.44 ± 0.04 b	0.30 ± 0.012 d
Red Salanova × 16 µM Se	1,349 ± 10 cd	15.5 ± 0.09	9.6 ± 0.15 d	54.89 ± 0.29 cd	5.51 ± 0.37	2.73 ± 0.05 a	0.35 ± 0.015 bcd
Red Salanova × 24 µM Se	1,334 ± 54 cd	14.6 ± 0.06	16.4 ± 0.51 b	56.70 ± 0.31 cd	5.32 ± 0.17	2.72 ± 0.01 a	0.36 ± 0.016 bcd
Red Salanova × 32 µM Se	1,293 ± 47 cd	12.6 ± 0.28	26.1 ± 1.56 a	58.53 ± 2.14 abc	5.30 ± 0.11	2.80 ± 0.03 a	0.57 ± 0.112 a
Red Salanova × 40 µM Se	1,103 ± 45 d	12.0 ± 0.40	27.6 ± 0.39 a	52.47 ± 1.48 d	4.68 ± 0.33	2.64 ± 0.15 a	0.48 ± 0.038 ab
	*	ns	***	*	ns	**	ns

ns,*,**, *** Non-significant or significant at P ≤ 0.05, 0.01, and 0.001, respectively. In the absence of interaction, cultivar means were compared by t-Test and Se application means by Duncan’s multiple-range test (P = 0.05). Different letters within each column indicate significantly different means. All data are expressed as mean ± SE, n = 3.

The growth and development of plants depends on the equilibrium of the mineral elements, as stress occurs in the presence of nutritional imbalances (Salt et al., 2008). Minerals are also essential for human health and lettuce is considered a good source of them (Baslam et al., 2013; Kim et al., 2016). Irrespective of Se concentration in the nutrient solution, green Salanova recorded the higher potassium and calcium content, while red Salanova showed the higher quantity of magnesium and sulphate (**Table 2**). As previously reported in literature, lettuce mineral content is quite variable depending on head type, leaf color and cultivar (Kim et al., 2016). However, regardless Se concentration in the nutrient solution and lettuce cultivar, our results particularly, potassium, calcium and magnesium were proximate to those reported by Blasco et al. (2012) on lettuce grown in controlled environment conditions.

Neither cultivar nor Se treatment had significant effect on Na accumulation in leaf tissue (avg. 0.37 g kg^−1^ dw), whereas phosphate and calcium were highly influenced by cultivar and Se concentration with no significant interaction between the two tested factors (**Table 2**). Averaged over cultivar, phosphate content decreased significantly (about 15%) in response to Se treatments from 24 to 40 µM compared to the 0 to 16 µM treatments. In addition, the calcium content at 40 µM Se was significantly lower than the control (9%) (**Table 2**). Our findings, are in line with those of Rios et al. (2013) who reported a 9% decrease in calcium concentration at a Se dose of 40 µM compared to the control and a similar reduction in phosphate content was also observed by the same authors in response to Se concentration ranging from 20 to 120 µM.

Leaf contents in potassium, magnesium and sulphate were influenced by cultivar and Se treatments with significant C × Se interaction (**Table 2**). In green Salanova, a significant reduction of K was observed at Se 8 µM (10%) and 40 µM (17%) compared to the control (**Table 2**). Likewise, a 10% decrease in Mg content was noted with respect to the control, both at 8 and 40 µM Se. On the contrary, in the red cultivar potassium content spiked by 9% at Se 32 µM and magnesium content by about 12% increase when Se treatment ranged between 16-40 µM, compared to the control treatment (**Table 2**). The lowest K and Mg contents observed in green Salanova at 40 µM Se application coincide with the results obtained by Rios et al. (2013) at the same dose of selenate on Philipus green lettuce cultivar. Similarly, Smoleń et al. (2016b) found a decrease in potassium content by about 9% in green butterhead lettuce leaves treated with selenium combined with iodine. On the other hand, the increase of K and Mg recorded in red Salanova treated with Se was in disagreement with other scientific literature where the authors found no variation in these two macroelements content after selenate applications (Wu and Huang, 1992; Silva et al., 2018a).

Furthermore, sulphate content increased significantly and linearly in both cultivars with selenate concentration ranging from 2.10 to 12.30 mg kg^−1^ dw in green Salanova and from 3.63 to 27.60 mg kg^−1^ dw in red Salanova (**Table 2**). These data imply a synergic relationship between selenate and sulphate. Selenium is chemically similar to sulfur, therefore plants absorb and metabolize Se *via* S uptake and assimilation pathway (Sors et al., 2005; Pilon-Smits and Quinn, 2010). Selenate is assimilated by plants through a process of active transport, which is driven by sulphate transporters (SULTR) (Dall’Acqua et al., 2019). SULTR mediate the movement of the sulfate in the vascular bundles, thus both selenate and sulphate are actively accumulated in the plant cells against their electrochemical gradient (Terry et al., 2000; Dall’Acqua et al., 2019). Our results are confirmed by White et al. (2004) who found that selenate applications promoted the accumulation of sulphate in the shoots of the model plant *Arabidopsis thaliana*. Similar findings were found in lettuce by several authors (Ramos et al., 2011; Hawrylak-Nowak, 2013; Rios et al., 2013; Silva et al., 2018a), and in particular Rios and co-workers (Ríos et al., 2008) reported an increase in S content in lettuce shoots with Se concentrations up to 40 µM. The first stage in the S-assimilation process consists of the activation of the enzyme ATP-sulfurylase, which produces adenosine phosphosulfate from sulfate and ATP (Pilon-Smits et al., 1999). Then, activated selenate is reduced *via* selenite to selenide and assimilated into SeCys and SeMet. These Se-amino acids can replace their S-analogues, amino acids Cys and Met in proteins (Sors et al., 2005; Van Hoewyk, 2013). In this sense, selenate applications can increase the ATP-sulfurylase activity and consequently a greater presence of selenate could imply increased production of Se and S end products (Ríos et al., 2008). Furthermore, despite the highest SULTR expression and sulphate translocation from roots to the shoots, certain S amino acids tend to decrease as the Se dosage increases. In *Eruca sativa* a lower leaf content of Cys and glutathione was found when plants were treated with Se concentrations equal to or higher than 10 µM (Dall’Acqua et al., 2019). It is conceivable that the lower accumulation of S-compounds may be due to the interference of Se with the S flow through the assimilation pathway, consequently reducing sulphate demand and eliciting a higher accumulation of this anion in the leaves.

**Figure 1:**
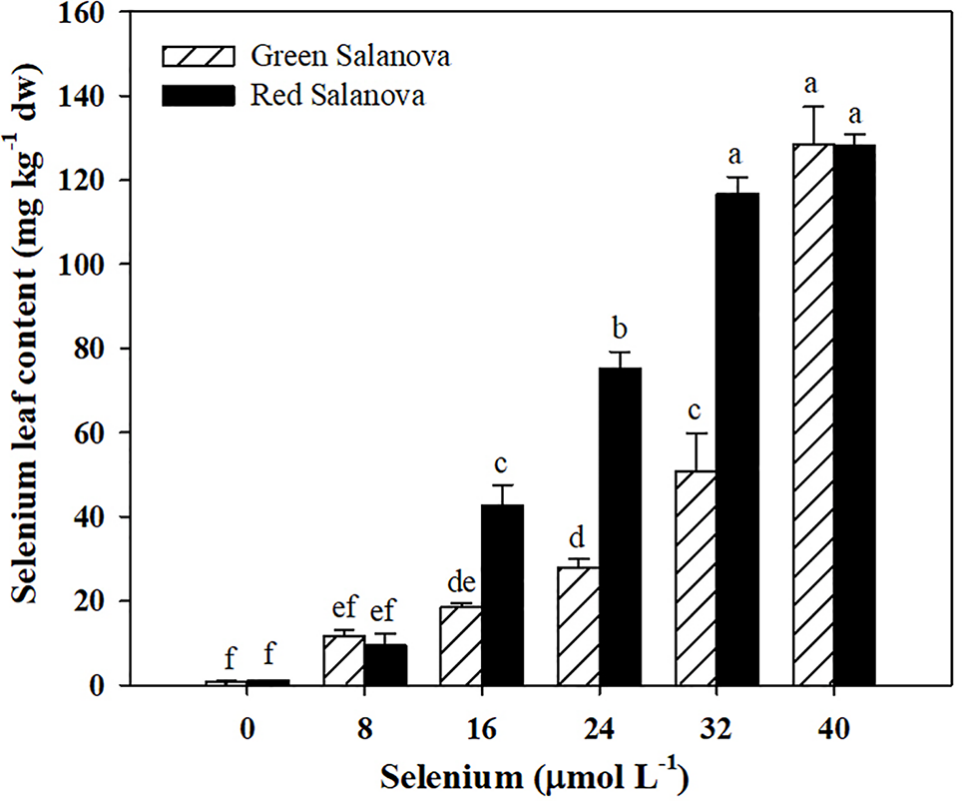
Effects of genotype and selenium concentration in the nutrient solution on selenium biofortification of green and red Salanova lettuce grown hydroponically in a Fitotron open-gas-exchange growth chamber under six Se concentrations applied in the nutrient solution. Different letters indicate significant differences according to Duncan’s test (P < 0.05). The values are means of three replicates. Vertical bars indicate ± SE of means.

The effectiveness of a selenium biofortification program is strongly related with the capacity of the candidate crop to assimilate and accumulate this element in the edible parts of the plant. In the current study Se leaf content increased with selenate application rate (**Figure 1**). Comparing cultivars, red leaf lettuce accumulated on average 57% more Se than green one. Selenium leaf content was influenced by cultivar and Se treatments with highly significant interaction between the two studied factors. In particular, Se concentration peaked in green Salanova at 40 µM dose (128.43 mg kg^−1^ dw), while in red Salanova it peaked at 32 and 40 µM (116.67 and 128.20 mg kg^−1^ dw of Se, respectively). Anyhow, Se leaf content was significantly higher than the control treatment in treatments ≥ 16 µM dose for both cultivars. Our results are in agreement with previous studies on red and green-pigmented lettuce (Ramos et al., 2010; Hawrylak-Nowak, 2013; Silva et al., 2018a) demonstrating the actual feasibility of using lettuce crop in Se biofortification programs.

In the Mediterranean basin, dietary habits vary according to geographical area, but overall the well-known Mediterranean diet is mainly based on cereals, fruit, vegetables, dairy products and meat. The daily intakes of food groups considered part of the Mediterranean diet are: 219 g of cereals, 247 g of fresh and dried fruit, 226 g of vegetables and legumes, 327 g of dairy products and 136 g of meat and fish (Couto et al., 2011). These food intakes, multiplied by the average Se concentration of the individual groups, correspond to a total Se intake of around 80 µg day^−1^ per capita. Considering that the RDA of this trace element stipulated for adults is 55 µg day^−1^ (Johnson et al., 2003), it can be deduced that Se deficiency has a very low incidence in the Mediterranean area. In other countries, such as Brazil, it was found that the Se intake is only 25 µg day^−1^, so about 30 µg Se day^−1^ must be integrated to reach the minimum recommended dose (Silva et al., 2019). The average serving of leafy vegetables, including lettuce, is about 50 g fw (Voogt et al., 2010). In our experiment, Se daily intake and percentage of RDA-Se for Se intake through consumption of 50 g portions of fresh green and red Salanova lettuce were influenced by cultivar and Se treatments with significant C × Se interaction (**Table 3**). Se daily intake increased significantly and linearly in both cultivars with selenate concentration ranging from 2 to 377 µg day^−1^ in green Salanova and from 4 to 355 µg day^−1^ in red Salanova (**Table 3**). Consequently, the RDA-Se varies with the same trend reaching a peak at 40 µM dose in both cultivars (685 and 646%, respectively for the green and red Salanova, respectively). Our RDA-Se values observed at the lowest Se dose (8 µM), were comparable with those found by Smoleń et al. (2019) on six varieties of lettuce biofortified with selenium combined with iodine at the 6.3 µM Se dose. Particularly, the iceberg varieties Krolowa and Maugli showed the lowest values (23.8 and 27.1%, respectively), while the green butterhead Cud Voorburgu and the red lettuce Lollo rossa reached the highest percentage (44.7 and 44.8%, respectively) which were comparable with the values found in green and red Salanova at the 8 µM Se dose (57 and 45%, respectively). Taking into account the Se biofortification target, 50 g fw day^−1^ of green and red Salanova at 16 µM Se dose provide 50 and 106 µg Se day^−1^ respectively (91 and 193% of the RDA), then in countries like Brazil, the RDA can be satisfied by consuming only 15 g fw day^−1^ of red Salanova or 30 g fw day^−1^ of green Salanova. On the other hand, in order to assess the risks to human health, the green vegetables hazard quotient (HQ_gv_) was calculated according to the United States Environmental Protection Agency (USEPA) Protocol, where HQ_gv_ values below 1.00 indicate that the vegetable is safe for consumption by human beings. In the current study HQ_gv_ increased with selenate application rate ranging from 0.00 to 0.94 in green Salanova and from 0.01 to 0.89 in red Salanova, therefore the 50 g daily portion of biofortified lettuce can be considered safe since the values of HQ_gv_ are less than 1 in all treatments (**Table 3**). In particular, in lettuce at 16 µM Se dose, the HQ_gv_ values are very low (0.12 and 0.27, respectively for green and red Salanova), indicating that even if the standard 50 g portion was tripled, these vegetables would not be in any case detrimental to human health.

**Table 3:** Selenium daily intake, percentage of recommended daily allowance for Selenium (RDA-Se) and hazard quotient (HQ_gv_) for Se intake through consumption of 50 g portions of fresh green and red Salanova lettuce by adult humans (70 kg body weight) grown hydroponically in a Fitotron open-gas-exchange growth chamber under six Se concentrations applied in the nutrient solution.

Source of variance	Se intake with 50 g fw of lettuce (μg day^−1^)	RDA-Se with 50 g fw of lettuce (%)	HQ_gv_ with 50 g fw of lettuce
Cultivar (C)			
Green Salanova	113 ± 31	205 ± 56	0.28 ± 0.1
Red Salanova	166 ± 33	302 ± 60	0.42 ± 0.1
t-test	ns	ns	**
Selenium (µM Se) (S)			
0	3 ± 0.5	5 ± 0.8	0.01 ± 0.0
8	28 ± 4.1	51 ± 7.4	0.07 ± 0.0
16	78 ± 14	142 ± 26	0.20 ± 0.0
24	136 ± 27	247 ± 49	0.34 ± 0.1
32	226 ± 41	410 ± 74	0.56 ± 0.1
40	366 ± 12	665 ± 21	0.91 ± 0.0
	***	***	***
C × S			
Green Salanova × 0 µM Se	2 ± 0.5 h	4 ± 0.8 h	0.00 ± 0.0 h
Green Salanova × 8 µM Se	31 ± 4.3 gh	57 ± 7.8 gh	0.08 ± 0.0 gh
Green Salanova × 16 µM Se	50 ± 1.0 fg	91 ± 1.8 fg	0.12 ± 0.0 fg
Green Salanova × 24 µM Se	77 ± 5.4 ef	139 ± 10 ef	0.19 ± 0.0 ef
Green Salanova × 32 µM Se	139 ± 22 d	253 ± 40 d	0.35 ± 0.1 d
Green Salanova × 40 µM Se	377 ± 24 a	685 ± 44 a	0.94 ± 0.1 a
Red Salanova × 0 µM Se	4 ± 0.3 h	7 ± 0.6 h	0.01 ± 0.0 h
Red Salanova × 8 µM Se	25 ± 7.3 gh	45 ± 13 gh	0.06 ± 0.0 gh
Red Salanova × 16 µM Se	106 ± 14 de	193 ± 25 de	0.27 ± 0.0 de
Red Salanova × 24 µM Se	195 ± 12 c	354 ± 22 c	0.49 ± 0.0 c
Red Salanova × 32 µM Se	312 ± 19 b	567 ± 34 b	0.78 ± 0.0 b
Red Salanova × 40 µM Se	355 ± 0.8 a	646 ± 1.4 a	0.89 ± 0.0 a
	***	***	***

ns,**, *** Nonsignificant or significant at P ≤ 0.01, and 0.001, respectively. In the absence of interaction, cultivar means were compared by t-Test and Se application means by Duncan’s multiple-range test (P = 0.05). Different letters within each column indicate significantly different means. All data are expressed as mean ± SE, n = 3.

### Target Phenolic Compounds and Carotenoids Profiles

HPLC analysis revealed in both cultivars the presence of four main caffeic acid derivatives (**Table 4**). Chicoric acid was the most abundant phenolic acid detected in both cultivars (101.44 and 105.99 mg 100 g^−1^ dw, respectively for the green and the red cultivar), chlorogenic acid (88.02 mg 100 g^−1^ dw) and caffeoyl-meso-tartaric acid (41.08 mg 100 g^−1^ dw) were higher in red Salanova, while caffeoyl-tartaric acid (17.77 mg 100 g^−1^ dw) was higher in green Salanova compared to the red cultivar (**Table 4**). The sum of detected phenolic acids was higher in the red-pigmented cultivar with respect to the green one (239.52 and 139.10 mg 100 g^−1^ dw, respectively). The content of phenolic acids varies according to the type of lettuce (Kim et al., 2016). Our results are consistent with the literature in which red cultivars have more phenolic acids than green ones (Llorach et al., 2008; Kim et al., 2016). The presence of chlorogenic acid, chicoric acid, and caffeoyl tartaric acid was also detected in seven different lettuce cultivars previously studied by Rouphael et al. (2017a). All phenolic acids were affected by cultivar and Se treatments with significant cultivar × Se interaction (**Table 4**). In green Salanova, caffeoyl-tartaric acid increased by 69% and 46% respectively at Se doses of 16 and 24 µM, but decreased by 75% at 32 µM, while in red Salanova the highest content was obtained at 16 µM (105%) compared to the control. Chorogenic acid in the green cultivar decreased by 57% at Se 32 µM but increased by 143% at the most concentrated Se dose, while in the red cultivar the content increased at 8, 16, 24 and 40 µM with the highest value recorded at 16 µM (191.64 mg 100 g^−1^ dw). Similarly, chicoric acid in the green cultivar increased at Se doses of 8, 16, 24 and 40 µM with the highest value recorded at 16 µM (148.53 mg 100 g^−1^ dw), but decreased by 67% at 32 µM; conversely, in the red cultivar chicoric acid content increased by 32% at 16 µM but decreased at Se doses 8, 24, 32 and 40 µM (**Table 4**). In red Salanova, caffeoyl-meso-tartaric acid increased by 270%, 84% and 89%, respectively, by adding in the nutrient solution 16, 24, and 40 µM of Se compared to the control treatment, while no significant differences were found for this phenolic acid in green Salanova. In the green cultivar, the sum of detected phenolic acids was significantly higher at 8, 16, 24, and 40 µM with the highest value observed at 24 µM (194.55 mg 100 g^−1^ dw), but decreased by 67% at 32 µM, while in red cultivar the sum of phenolic acids increased by 112% at 16 µM and decreased at Se doses of 8, 32, and 40 µM compared to the control (**Table 4**).

**Table 4:** Phenolic acids composition, total phenolic acids and anthocyanins of green and red Salanova lettuce grown hydroponically in a Fitotron open-gas-exchange growth chamber under six Se concentrations applied in the nutrient solution.

Source of variance	Caffeoyl tartaric acid (mg 100 g^−1^ dw)	Chlorogenic acid (mg 100 g^−1^ dw)	Chicoric acid (mg 100 g^−1^ dw)	Caffeoyl meso tartaric acid (mg 100 g^−1^ dw)	∑ phenolic acids (mg 100g^−1^ dw)	Anthocyanins (μg cyanidin eq. g^−1^ dw)
Cultivar (C)						
Green Salanova	17.77 ± 1.86	13.94 ± 1.51	101.44 ± 9.27	5.96 ± 0.49	139.10 ± 12.42	n.d.
Red Salanova	4.43 ± 0.42	88.02 ± 11.71	105.99 ± 12.20	41.08 ± 5.11	239.52 ± 26.73	13.28 ± 1.45
t-test	***	***	ns	***	**	–
Selenium (µM Se) (S)						
0	9.99 ± 2.76	30.76 ± 9.46	116.65 ± 16.75	14.59 ± 3.50	171.99 ± 25.77	8.76 ± 0.23 d
8	11.79 ± 3.48	45.34 ± 14.22	92.41 ± 8.03	16.16 ± 4.47	165.70 ± 8.33	8.73 ± 0.37 d
16	17.56 ± 4.43	103.47 ± 39.47	160.34 ± 15.88	44.45 ± 17.67	325.81 ± 68.1	24.85 ± 2.58 a
24	13.97 ± 4.48	51.68 ± 15.67	114.71 ± 15.29	23.31 ± 8.09	203.68 ± 6.37	16.10 ± 0.96 b
32	3.42 ± 0.34	28.67 ± 11.01	45.83 ± 7.90	17.21 ± 6.82	95.13 ± 25.33	11.48 ± 0.56 c
40	9.88 ± 2.89	45.96 ± 10.37	92.33 ± 9.29	25.39 ± 7.73	173.56 ± 8.48	9.78 ± 0.39 cd
	***	***	***	***	***	***
C x S						
Green Salanova × 0 µM Se	16.15 ± 0.27 c	9.76 ± 0.97 g	85.40 ± 3.40 d	6.85 ± 0.23 d	118.17 ± 3.82 g	n.d.
Green Salanova × 8 µM Se	19.30 ± 1.98 c	13.71 ± 1.46 g	109.83 ± 4.00 c	6.36 ± 0.19 d	149.19 ± 6.72 f	n.d.
Green Salanova × 16 µM Se	27.23 ± 2.09 a	15.30 ± 1.18 fg	124.90 ± 1.53 c	6.33 ± 0.70 d	173.75 ± 2.52 def	n.d.
Green Salanova × 24 µM Se	23.60 ± 2.67 b	16.99 ± 0.64 fg	148.53 ± 4.47 b	5.43 ± 0.70 d	194.55 ± 7.59 cd	n.d.
Green Salanova × 32 µM Se	4.00 ± 0.37 e	4.18 ± 0.66 h	28.35 ± 1.47 f	2.21 ± 0.41 d	38.74 ± 2.31 h	n.d.
Green Salanova × 40 µM Se	16.32 ± 0.45 c	23.73 ± 0.62 f	111.63 ± 7.62 c	8.55 ± 0.39 d	160.23 ± 8.46 ef	n.d.
Red Salanova × 0 µM Se	3.84 ± 0.06 e	51.76 ± 2.26 e	147.89 ± 20.38 b	22.32 ± 1.10 c	225.82 ± 20.25 b	8.76 ± 0.23
Red Salanova × 8 µM Se	4.27 ± 0.12 de	76.98 ± 2.90 c	75.00 ± 1.79 de	25.96 ± 1.93 c	182.21 ± 5.41 de	8.73 ± 0.37
Red Salanova × 16 µM Se	7.89 ± 0.63 d	191.64 ± 3.96 a	195.78 ± 1.65 a	82.57 ± 10.34 a	477.87 ± 7.83 a	24.85 ± 2.58
Red Salanova × 24 µM Se	4.34 ± 0.72 de	86.38 ± 4.79 b	80.90 ± 2.22 de	v ± 2.69 b	212.80 ± 7.87 bc	16.10 ± 0.96
Red Salanova × 32 µM Se	2.84 ± 0.32 e	53.16 ± 2.48 e	63.31 ± 2.10 e	32.21 ± 2.69 bc	151.52 ± 4.75 f	11.48 ± 0.56
Red Salanova × 40 µM Se	3.43 ± 0.19 e	68.18 ± 6.56 d	73.04 ± 1.02 de	73.04 ± 3.80 b	186.89 ± 10.49	9.78 ± 0.39
	***	***	***	***	***	–

ns, **, *** Nonsignificant or significant at P ≤ 0.01, and 0.001, respectively. In the absence of interaction, cultivar means were compared by t-Test and Se application means by Duncan’s multiple-range test (P = 0.05). Different letters within each column indicate significantly different means. All data are expressed as mean ± SE, n = 3. n.d. not detectable.

Our results showed irregular variation of phenolic acids content in both cultivars, as the concentrations of these hydrophilic antioxidant molecules varied with Se concentration without a clear trend. Furthermore, this pattern is consistent with what was found by Schiavon et al. (2016) in radish and by D’Amato et al. (2018) in rice sprouts, but is in disagreement with Ríos et al. (2008) who reported a rise in the total phenol content of lettuce as the Se dose applied increased. On the other hand, the presence of Se constitutes an abiotic stress similar to that caused by other heavy metals. Plants react to their presence by activating the phenylpropanoid pathway (Wang et al., 2016) to produce phenolic compounds that can chelate metals and inhibit enzymes such as xanthine oxidase in an effort to prevent the production of Reactive Oxygen Species (ROS) (Ríos et al., 2008).

Anthocyanins are one of the phenolic phytochemical subclasses (Harborne and Williams, 2001) encompassing water-soluble pigments responsible for the red pigmentation in lettuce (Kim et al., 2016). Consequently, these pigments were not detected in green Salanova but exclusively in the red cultivar with an average concentration of 13.28 µg cyanidin eq. g^−1^ dw (**Table 4**). Anthocyanins have many physiological effects on plants and humans, such as antioxidation, protection against ultraviolet damage and the prevention and treatment of various diseases (Hamilton, 2004). Anthocyanins in red Salanova, were found to be significantly affected by selenate applications; in particular they increased by 184%, 84%, and 31% respectively at Se doses of 16, 24, and 32 µM compared to the control (**Table 4**). Our results are in accordance with Liu et al. (2017), where anthocyanins in red lettuce cv. Purple Rome increased significantly at moderate doses of Se, while they were lower and comparable to the control at higher Se doses. In their study, the authors showed that the Se influence on accumulation and molecular regulation of anthocyanins synthesis was mainly due to the expression levels of the flavanone 3-hydroxylase (F3H) and UDP-glycose flavonoid glycosyl transferase (UFGT) genes that played a key role in anthocyanins biosynthesis. The F3H and UFGT genes were significantly up-regulated by moderate Se treatments compared to the control (Liu et al., 2017).

Carotenoids are essential lipid-soluble pigments that have antioxidant properties and are found in all photosynthetic organisms (Gross, 1991). These compounds play significant roles in the prevention of chronic ailments, such as cancer, cardiovascular disease, diabetes and osteoporosis, owing to their potent antioxidant, immunomodulatory, gap-junction communication, photoprotective, neuroprotective, and vitamin A activity (Saini et al., 2015). Carotenoids are classified into two groups, xanthophylls which include neoxanthin, violaxanthin, lutein, zeaxanthin, and β-cryptoxanthin, and carotenes which include β-carotene, α-carotene, and lycopene. In human diet, neoxanthin, violaxanthin, lutein, and β-carotene are primarily obtained from dark green or red vegetables. Specifically in lettuce, higher carotenoids content has been found in red leaf cultivars compared to green ones (Nicolle et al., 2004). This finding is in agreement with our results where red Salanova had a significantly higher content of all the target carotenoids detected compared to green Salanova. The sum of all detected carotenoids was 133% higher in the red cultivar compared to the green one (**Table 5**). As in the case of phenolic compounds, the content in target carotenoids was affected by both cultivar and Se treatments with significant however cultivar × Se interaction (**Table 5**). In green Salanova, all detected carotenoids decreased in response to selenate applications compared to the control (**Table 5**), whereas in red Salanova this trend was differentiated. violaxanthin + neoxanthin, lutein, and β-cryptoxanthin increased in red Salanova with increasing selenate application levels, reaching their highest levels at the 32 µM Se dose, whereas β-carotene in the 24–40 µM Se dose range was on average 23% lower than the control. Regarding the green cultivar, our results are in agreement with what has been found in the literature on lettuce (Hawrylak-Nowak, 2013), rice (D’Amato et al., 2018), and *Arabidopsis* (Sams et al., 2011), where a reduction of the total carotenoids content was observed following the application of sodium selenate. Pertinent to these results is previous work on *Arabidopsis* that has demonstrated that the presence of selenate may down-regulate phytoene synthase, a major enzyme involved in the biosynthesis of carotenoids (Sams et al., 2011). On the other hand, the increase in xanthophylls (violaxanthin + neoxanthin, lutein and β-cryptoxanthin) found in red Salanova in response to Se doses up to the 32 µM could be associated to a dissimilar activation of molecular and physiological mechanisms in this cultivar, which differently influence the biosynthesis and accumulation of secondary metabolites, such as xanthophylls. Moreover, in our experiment, it was noted that the presence of selenate had contrasting effects on various classes of secondary metabolites.

**Table 5:** Composition of carotenoids profile of green and red Salanova lettuce grown hydroponically in a Fitotron open-gas-exchange growth chamber under six Se concentrations applied in the nutrient solution.

Source of variance	Violaxanthin + neoxanthin (μg violaxanthin eq. g^−1^ dw)	Lutein (μg g^−1^ dw)	β-Cryptoxanthin (μg g^−1^ dw)	β-carotene (μg g^−1^ dw)
Cultivar (C)				
Green Salanova	507.39 ± 14.1	207.62 ± 8.55	370.60 ± 13.8	165.62 ± 6.53
Red Salanova	993.13 ± 28.8	600.36 ± 15.3	989.43 ± 26.4	337.14 ± 11.8
t-test	***	***	***	***
Selenium (µM Se) (S)				
0	733.14 ± 53.0	421.04 ± 62.6	717.66 ± 107	296.43 ± 37.0
8	633.57 ± 95.3	357.59 ± 81.6	587.32 ± 127	252.25 ± 51.7
16	774.82 ± 117	421.51 ± 101	699.87 ± 165	272.02 ± 57.1
24	762.72 ± 123	385.30 ± 88.9	645.43 ± 138	215.09 ± 29.6
32	850.46 ± 148	461.27 ± 113	784.17 ± 176	239.98 ± 33.2
40	746.85 ± 118	377.20 ± 81.1	645.67 ± 119	232.51 ± 23.2
	***	***	***	***
C × S				
Green Salanova × 0 µM Se	614.93 ± 5.54 d	282.15 ± 3.01 e	478.51 ± 3.85 e	214.60 ± 5.39 e
Green Salanova × 8 µM Se	421.46 ± 7.09 f	175.52 ± 3.87 g	305.07 ± 5.49 h	136.91 ± 2.42 h
Green Salanova × 16 µM Se	513.05 ± 3.29 e	195.75 ± 4.01 fg	331.35 ± 6.79 gh	145.04 ± 3.10 gh
Green Salanova × 24 µM Se	489.24 ± 7.10 e	186.75 ± 2.57 fg	337.96 ± 8.31 gh	149.09 ± 2.93 gh
Green Salanova × 32 µM Se	520.97 ± 4.26 e	209.40 ± 5.19 f	390.71 ± 2.76 f	166.05 ± 4.61 fg
Green Salanova × 40 µM Se	484.69 ± 2.68 e	196.11 ± 3.01 fg	379.99 ± 6.92 fg	182.06 ± 2.73 f
Red Salanova × 0 µM Se	851.34 ± 6.70 c	559.94 ± 17.4 cd	956.81 ± 21.7 c	378.27 ± 10.1 ab
Red Salanova × 8 µM Se	845.68 ± 19.1 c	539.67 ± 10.4 d	869.57 ± 32.3 d	367.60 ± 8.28 b
Red Salanova × 16 µM Se	1,036.59 ± 11.4 b	647.27 ± 15.1 b	1,068.38 ± 25.7 b	399.01 ± 13.9 a
Red Salanova × 24 µM Se	1,036.19 ± 17.1 b	583.85 ± 7.42 c	952.89 ± 8.83 c	281.09 ± 3.13 d
Red Salanova × 32 µM Se	1,179.95 ± 20.8 a	713.14 ± 0.18 a	1,177.62 ± 26.2 a	313.91 ± 4.53 c
Red Salanova × 40 µM Se	1,009.02 ± 26.4 b	558.28 ± 10.9 cd	911.34 ± 16.9 cd	282.95 ± 11.8 d
	***	***	***	***

ns, *** Non-significant or significant at P ≤ 0.001, respectively. In the absence of interaction, cultivar means were compared by t-Test and Se application means by Duncan’s multiple-range test (P = 0.05). Different letters within each column indicate significantly different means. All data are expressed as mean ± SE, n = 3.

### Principal Component Analysis

**Figure 2:**
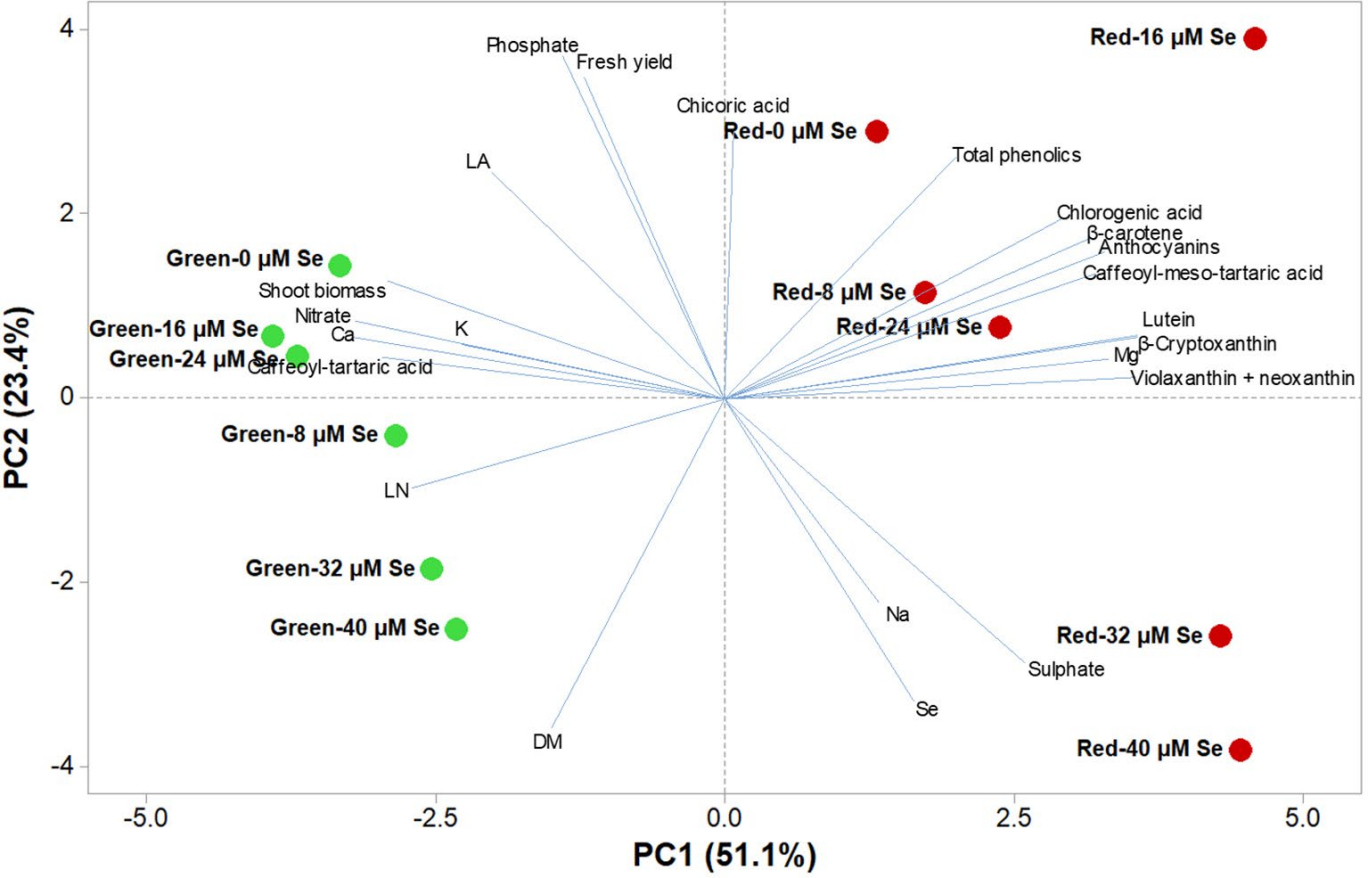
Principal component loading plot and scores of principal component analysis (PCA) of growth parameters (leaf area: LA and leaf number: LN), fresh yield, shoot dry biomass mineral concentrations (Nitrate, phosphate, sulphate, K, Ca, Mg, and Na), lipophilic and hydrophilic antioxidant molecules (target phenolic acids and total phenolics, anthocyanins, ascorbic acid and target carotenoids) in green and red butterhead lettuce Salanova grown under six different concentrations of selenium (Se) added as sodium selenate (0, 8, 16, 24, 32, and 40 μM).

A comprehensive overview of the nutritional and functional quality profiles determined by ion chromatography and HPLC-DAD on red and green butterhead Salanova lettuce in response to Se concentration in the nutrient solution was obtained through Principal Component Analysis (PCA; **Figure 2**). The principle component (PC1) accounted for 51.1% of the cumulative variance, while PC2, and PC3 explained 23.4 and 8.2%, respectively of the total variance (**Table 6**). PC1 correlated positively to the four target carotenoids, caffeoyl-meso-tartaric and chlorogenic acid, magnesium, and sulphate content. PC1 correlated negatively to agronomical traits (shoot biomass and leaf number), as well as to nitrate, calcium, and potassium content. PC2 positively correlated to fresh yield, chicoric acid, total phenolic acids, and phosphate content; and negatively to leaf dry matter and Se content (**Table 6**). Furthermore, the loading matrix indicated the correlations among the examined quanti-qualitative traits, wherein two variables at an angle < 90° were positively correlated, whereas an angle > 90° designated negatively correlated variables. In our experiment, variation in chlorogenic and anthocyanin contents were most closely aligned with β-carotene content, whereas variation in total phenolics did not correlate to nitrate content (**Figure 2**).

**Table 6:** Eigen values, relative and cumulative proportion of total variance, and correlation coefficients for growth parameters, mineral profile, nutritional and functional traits of Salanova butterhead lettuce with respect to the three principal components.

Principal components	PC1	PC2	PC3
Eigen value	11.7	5.3	1.8
Percentage of variance	51.1	23.4	8.2
Cumulative variance	51.1	74.5	82.7
Eigen vectors^a^			
Lutein	**0.957**	0.160	0.168
β-Cryptoxanthin	**0.956**	0.156	0.148
Violaxanthin + neoxanthin	**0.954**	0.057	0.240
Mg	**0.889**	0.101	0.363
Anthocyanins	**0.882**	0.370	**−**0.044
Ca	**−0.858**	0.154	0.236
Caffeoyl-meso-tartaric acid	**0.858**	0.315	**−**0.113
Nitrate	**−0.855**	0.198	0.362
β-carotene	**0.850**	0.410	**−**0.049
Caffeoyl-tartaric acid	**−0.790**	0.109	**−**0.024
Shoot biomass	**-0.781**	0.300	**−**0.007
Chlorogenic acid	**0.781**	0.452	**−**0.206
LN	**−0.724**	**−**0.219	**−**0.347
Sulphate	**0.697**	**−0.657**	0.108
Phosphate	**−**0.374	**0.860**	0.187
DM	**−**0.399	**−0.820**	**−**0.293
Fresh yield	**−**0.323	**0.808**	0.216
Se	0.440	**−0.755**	**−**0.187
Chicoric acid	0.019	**0.672**	−0.374
Total phenolics	0.535	**0.609**	−0.298
LA	**−**0.540	0.571	−0.218
K	**−0.608**	0.139	**0.676**
Na	0.359	**−**0.507	0.586

a Boldface factor loadings are considered highly weighed.

b LN, leaf number; DM, dry matter; LA, leaf area.

The effectiveness of PCA in interpreting cultivar differences across multiple nutritional and functional quality characters in response to several pre-harvest factors (e.g., nutrient solution management, biofortification, plant biostimulants) has been previously demonstrated (Colonna et al., 2016; Cardarelli et al., 2017; El-Nakhel et al., 2019). This was also the case in our study, since the score plot of the PCA highlighted crucial information on the nutritional and functional quality of the tested butterhead cultivars exposed to different Se concentrations in the nutrient solution. The PCA clearly divided the two tested cultivars along PC1 with red-pigmented lettuce on the positive side and the green one on the negative side. Accordingly, green-pigmented lettuce distinguished for fresh and dry biomass, nitrate and mineral profile (Ca, phosphate and K contents); whereas the red-pigmented cultivar was superior in target lipophilic and hydrophilic antioxidant molecules as well as in total phenolic acids (**Figure 2**). Particularly, the red-pigmented lettuce treated with 8, 16, and 24 µM Se, positioned in the upper right quadrant of the PCA score plot, delivered premium quality and high concentration of hydrophilic and lipophilic antioxidants (**Figure 2**). Red Salanova at the highest two doses of Se was characterized by high content of Se and sulphate. Green butterhead lettuce grown under 0, 16, and 24 µM Se was positioned in the upper left quadrant, characterized overall by higher plant growth parameters (leaf area, fresh yield and shoot dry biomass) and mineral composition (phosphate, K, and Ca). Finally, the lower left quadrant depicted high Se concentration treatments of green lettuce, which yielded the lowest nutritional and functional quality traits of all 12 treatments except from a high percentage of leaf dry matter content (**Figure 2**). The PCA performed in the present study configured an integrated view of yield and quality traits quantitated by ion chromatography and HPLC. It thus enabled the interpretation of variation patterns in these traits with respect to the genetic material and Se biofortification applications studied.

## Conclusions

As demand for functional foods with beneficial effects on human health is rising, selenium biofortification of lettuce facilitated in closed soilless cultivation is presently demonstrated as an effective, low-cost method to produce Se-enriched food of high nutritional value. Our findings indicate that shoot dry biomass, mineral composition, as well as phenolic acids and carotenoids were strongly affected by genotype, with the red cultivar proved to have higher nutritional and functional quality than the green one. Our results demonstrated that the application of 16 µM Se in the nutrient solution improved the phenolic acids content in both cultivars, especially in red Salanova, which was also distinguished by a substantial increase in anthocyanins content (184%). In green Salanova, Se applications slightly reduced the overall carotenoids content, while in the red cultivar 16 and 32 µM Se doses triggered an increase in violaxanthin + neoxanthin, lutein and β-cryptoxanthin. Therefore, we can deduce that the optimal Se dose is 16 µM, as it improves the nutraceutical characteristics in both cultivars with a slight and acceptable reduction in fresh marketable yield (8%) recorded only in green Salanova. Selenium leaf content increased significantly with the sodium selenate application rate in both cultivars. Moreover, the 16 µM treatment yielded sufficient Se leaf content to satisfy 91% and 193% of RDA of this trace element by consuming respectively 50 g fw of green and red Salanova, without any toxic effect to humans, since the amount does not exceed the maximum allowable intake.

## Data Availability Statement

The datasets generated for this study are available on request to the corresponding author.

## Author Contributions

AP wrote the first draft of the manuscript, followed the statistical analysis, and contributed to results data interpretation. CE-N carried out the Fitotron experiment and wrote the first draft of the manuscript. MG and SS performed the mineral analysis and data interpretation. MK and SP were involved in data analysis, data interpretation, and editing the manuscript. YR coordinated the whole project, provided the intellectual input, set up the experiment, and corrected the manuscript.

## Conflict of Interest

The authors declare that the research was conducted in the absence of any commercial or financial relationships that could be construed as a potential conflict of interest.
